# Supplementary material for: Are carbon emissions trading and green financial instruments synergistic? -Comprehensive quantitative research based on content analysis
Source: PLoS One. 2024 Mar 7;19(3):e0298601. doi: 10.1371/journal.pone.0298601 (PMC10919601; doi:10.1371/journal.pone.0298601)
Supplement: S1 Data — (ZIP) [file pone.0298601.s001.zip › Data/地级市Z_F工作报告.zip!/╔╜╢1⁄2/┴─│╟╩╨/└┤╫╘╣1⁄2╓┌║┼í╛┬φ┐╦╩2╛▌═°í┐_2008.docx]

各位代表：

现在，我代表市人民政府向大会作政府工作报告，请予审议，并请市政协委员和其他列席会议的同志提出意见。

一、过去五年工作回顾

市十四届人大一次会议以来，本届政府在省委、省政府和市委的正确领导下，坚持以邓小平理论和“三个代表”重要思想为指导，深入贯彻科学发展观，认真落实市十次、十一次党代会和市人大及其常委会各项决议，解放思想、抢抓机遇、团结实干、奋力开拓，胜利完成“十五”计划和“十一五”规划前两年任务，全市改革开放和现代化建设取得新的巨大成就。

——预计生产总值达到1000亿元左右，是2002年的2倍以上，五年年均增长16%以上。

——预计财政总收入达到93.6亿元，其中地方财政收入42.2亿元，是2002年的2.5倍。

——预计社会消费品零售总额310亿元，是2002年的2倍。

——预计城镇居民人均可支配收入12460元，是2002年的2倍，农民人均纯收入4500元，是2002年的1.8倍；城乡居民本外币储蓄余额657亿元，是2002年的1.94倍。

——政治文明、精神文明建设进一步加强，全市呈现政通人和、团结和谐的良好局面。

五年来，我们主要做了以下六个方面的工作：

（一）认真贯彻国家宏观调控政策。坚持以科学发展观统领全局，坚决贯彻落实中央和省委、省政府一系列决策部署，努力推动发展方式转变。严格落实党的各项支农惠农政策，粮食总产连续4年创历史最高水平，先后登上80亿、90亿斤两个大的台阶。着力优化投资结构，加大对重点领域的有效投入，五年完成全社会固定资产投资1600亿元。加快创新型城市建设步伐，建成国家级工程技术研究中心1家、国家级技术中心3家，争取国家“863”计划相关项目10项。高度重视并切实抓好节能减排工作，2007年主要污染物化学需氧量、二氧化硫排放量分别比2005年降低1.61%和1.69%，单位生产总值能耗、规模以上工业万元增加值能耗有望完成目标任务。

（二）着力调整优化经济结构。按照“一产调优、二产调强、三产做大”的思路，坚持不懈地推进经济结构战略性调整，预计全市三次产业比例调整为15.6∶59.2∶25.2，二三产业比重提高11个百分点。大力发展高效农业，林牧渔业产值占农业总产值的比重提高4.5个百分点，省级以上农业产业化龙头企业达到17家，标准化农产品生产基地面积达到243.7万亩。大力实施十大百亿产业和“5513”工程，工业化进程明显加快，预计规模以上工业企业个数是2002年的3.7倍、增加值3.4倍、主营业务收入5.8倍、利税5.7倍、利润6.4倍；高新技术产业产值所占比重达到22.76%，提高9.96个百分点；新增中国名牌10个、中国驰名商标7枚。大力发展旅游、商贸流通等产业，预计2007年服务业增加值比2002年增加152亿元。

（三）大力推进改革开放。国有资产管理体制改革和国有企业改革取得新进展，东阿阿胶、银河纸业等成功实现产权并购，三家上市公司成功实现股权分置改革。市级国库集中支付制度改革全面推开，国有粮食购销企业改革全面完成。农村综合改革扎实推进。城市管理执法体制改革迈出实质性步伐。非公有制经济快速发展，增加值占GDP的比重达到50%。努力深化改革、扩大开放，五年累计引进项目到位资金579亿元。信发铝电、祥光铜业、天沐温泉等一大批项目建成投产。对外合作交流不断扩大，缔结国际友好城市4个，累计利用外资5.34亿美元，2007年进出口总额预计达到16.5亿美元，其中出口9.5亿美元，是2002年的6.8倍。

（四）全面加强城市和新农村建设。以打造卓越秀美的江北水城为目标，加强城市规划建设管理，城市形象和知名度大幅提升。新一轮城市总体规划修编工作全面完成，控制性详规覆盖率达到100%。城市开发建设累计投资183亿元，其中聊城城区90亿元，建设了水城明珠大剧场、中国运河文化博物馆等一批精品工程，新增城市绿地面积478公顷，新增城市道路498万平方米。积极开展“五城同创”，成功创建中国优秀旅游城市、国家卫生城和省级园林城市，创建国家环保模范城通过了国家技术核查，创建省级文明城市取得重要进展。城镇化率由2002年的26.1%提高到39.5%。县市城区和小城镇建设取得重大进展，基本实现了村村通油路、村村通客车，自来水普及率达到83%，通有线电视的行政村达到54%，沼气用户达到2.83万户，实现了城乡电网同网同价。基本建成了全市“半小时经济圈”。

（五）积极推进和谐社会建设。努力拓宽就业渠道，五年累计新增城镇就业再就业26.3万人，登记失业率控制在4%以下。劳务输出达到118万人。健全社会保障体系，养老保险金、失业救济金发放率均为100%；城市低保实现了应保尽保，标准逐年提高；农村低保制度初步建立。建立五保财政供养制度，新建改建农村中心敬老院40处，50%的农村五保对象实现集中供养。加大了对欠发达县、经济弱乡镇和莘县马西地区的帮扶力度。大力发展教育事业，建立了“以县为主”的义务教育管理体制和农村义务教育经费保障机制，新建改建高标准中小学校舍100多万平方米，全市累计有19.7万名毕业生升入大中专院校。高等教育快速发展，职业、成人和民办教育水平进一步提高。重视发展卫生事业，抗击非典取得重大胜利，公共卫生“两个体系”和动物疫病防控体系基本建成，新建改建乡镇卫生院108所，379万农民参加合作医疗，参合率达到92.2%。低生育水平持续稳定，人口自然增长率平均为4.92‰。积极发展文化事业，新编历史剧《大明贤后》荣获第十届中国戏剧节三项大奖。成功举办了五届江北水城文化旅游节、四届水城之秋观光周和第一届市运会。扎实推进平安聊城建设，切实抓好安全生产，加强社会治安综合治理，保持了社会和谐稳定。国防动员、人民防空、民兵预备役工作取得新成绩，连续两次被评为“全国双拥模范城”。新闻出版、广播电视、社会科学、外侨、对台、档案、妇女儿童、老龄、残疾人、民族、宗教、史志、地震、气象等工作都取得新的进展。

（六）切实加强政府自身建设。坚持依法行政，大力推进政府职能转变，深化行政审批制度改革，精简行政许可项目300项。坚持科学民主依法决策，设立了市长信箱、市长公开电话，建立了人民建议征集制度，重大决策听取社会各界意见。大力推进政务公开，加强电子政务建设，开通了政府网站，实现了市级电子公文传输。坚持从严治政，深入开展解放思想大讨论活动，认真解决工作中存在的矛盾和问题，提高政府的公信力和执行力。切实加强廉政建设和反腐败工作，查处了一批违法违纪案件。

各位代表！五年来，聊城的发展变化是巨大的。这五年，是我市经济社会又好又快发展、综合经济实力显著增强的五年，是城乡面貌发生巨大变化、聊城形象提升最快的五年，是人民群众收入增加最快、得到实惠最多的五年，是在全面建设小康社会、建设强市名城的道路上阔步前进的五年！

各位代表，五年来，中央和省领导同志多次视察聊城，指导工作，特别是胡锦涛总书记2003年12月来聊城视察，对聊城的发展提出殷切希望，给全市人民极大的鼓舞和鞭策。回顾五年历程，我们深深感到，五年来成绩的取得，是认真执行党中央、国务院和省委、省政府一系列指示精神的结果，是市委坚强领导的结果，是各级人大、政协和社会各界大力支持的结果，是全市人民同心同德、干事创业的结果。在此，我代表市人民政府,向全市工人、农民、知识分子，向关心支持政府工作的各民主党派、工商联、无党派人士和社会团体，向驻聊人民解放军、武警官兵、公安干警和中央、省驻聊单位，向所有关心和支持我市发展的海内外朋友，向全市所有的纳税人，表示衷心的感谢和崇高的敬意！

各位代表！在肯定成绩的同时，我们也必须清醒地认识到，我市经济社会发展还存在不少问题和困难，政府工作也存在不少缺点和不足。经济方面：总量较小，结构性矛盾比较突出，投资强度不够，发展后劲不足，农民持续增收难度较大，对外开放水平不高，节能减排压力很大。社会方面：受经济发展水平的制约，教育、卫生、文化、体育、就业、社保、住房等难以完全满足人民群众日益增长的需求。环境方面：从根本上治理“三乱”的体制机制尚未建立，吃拿卡要的问题还时有发生。人才短缺和人才浪费的现象同时存在。自身建设方面：政府职能还需进一步转变，铺张浪费现象在个别地方和单位还不同程度地存在等。对存在的问题，我们要高度重视，采取有力措施，切实加以解决。

二、今后五年工作总体要求

今后五年，是聊城推进新型工业化、城镇化的爬坡阶段，是全面建设小康社会、向基本实现现代化迈进的战略机遇期。政府工作的总体要求是：全面贯彻落实党的十七大精神，以科学发展观为统领，紧紧围绕建设强市名城实现新跨越的总目标，始终坚持解放思想、抢抓机遇，好中求快、又好又快，统筹发展、富民优先，立足当前、面向未来四项原则要求，突出抓好科学发展、三农稳市、工业强市、三产兴市、城建靓市、改革开放、和谐建设七大工作重点，在全面建设小康社会的征程上迈出坚实步伐，开创改革开放和现代化建设的新局面！

今后五年的任务目标是：

——经济总量翻一番。全市生产总值年均增长15%，五年翻一番，突破2000亿元；地方财政收入年均增长16％以上，达到90亿元，翻一番，力争突破100亿元；全社会固定资产投资年均增长20%，翻一番，突破1000亿元；社会消费品零售总额年均增长15%以上，翻一番，突破600亿元。三次产业结构调整为10∶60∶30。高新技术产业产值比重年均提高2个百分点以上。

——城乡建设靓品牌。“江北水城”品牌在国内外的知名度更高、影响力更大、对区域的辐射带动力更强。突出抓好“两城一河”，启动古城区保护开发，着力打造全国著名“水中古城”；加快建设经济开发区工业新城，形成全市新的经济增长极；搞好徒骇河开发，打造独具特色的城市风情带；加大城中村改造力度，改善居民居住条件。城乡面貌焕然一新，初步建成环境优美、生态良好的宜居城市，城镇化率达到45%以上。

——改革开放迈大步。国企改革取得新突破，财税改革呈现新局面，机构改革取得新进展，政府职能实现新转变，对外开放迈出新步伐。民营经济增加值年均增长20%以上，突破1700亿元，进出口总值年均增长22%，达到45亿美元，直接利用外资年均增长15%左右。

——文化实现大发展。致力于提高全社会文明素质，文化大市建设取得重大进展。社会主义核心价值体系深入人心，全民思想道德和文化素质不断提高，公共文化服务体系基本建立，文化产业占国民经济比重明显提高，文化软实力显著增强。

——人民生活上台阶。城镇居民人均可支配收入年均增长8%左右，农民人均纯收入年均增长10％左右。教育、卫生、体育、就业、社保等各项事业迈上新台阶；社会治安和安全生产形势稳定；精神文明和民主法制建设取得新进展。每年人口自然增长率控制在6.5‰以内，城镇登记失业率控制在4%以内。人民群众的安全感、幸福感、满意度明显提高。

实现五年的奋斗目标，关键是第一年。今年的预期目标是：全市生产总值增长15%；地方财政收入增长20%，突破50亿元；全社会固定资产投资增长22%；高新技术产业产值比重达到25%；利用外商直接投资增长20%；外贸进出口增长25%；单位生产总值能耗下降4.7%，化学需氧量排放降低7%，二氧化硫排放量减少3.5%；社会消费品零售总额增长16%；城镇居民人均可支配收入增长10%，农民人均纯收入增长10%。

各位代表！实现上述目标，任务光荣而艰巨。在前进的道路上，我们肯定会遇到许多困难、矛盾和挑战，但是，我们更面临前所未有的发展机遇。党的十七大胜利召开，为我们指明了前进方向；国家继续加强和改善宏观调控，为我市加快发展提供了良好的发展环境；省委、省政府“一体两翼”的发展战略，为我市加快发展提供了有利的外部条件；经过全市人民多年的不懈努力，我市经济社会发展已经站在新的起点上，具备了更好更快发展的基础和条件。特别是全市人民日益增强的自信心、自豪感和不断迸发的进取意识和创业激情，必将转化为又好又快发展的强大动力。只要我们咬定目标、振奋精神、开拓进取、负重奋进，聊城就一定能够在经济社会发展上迈出新的更大的步伐！就一定能够以更加昂扬的斗志、更加辉煌的业绩屹立于鲁西大地！聊城人民就一定能够过上更加富裕文明、更加和谐安定的幸福生活！

三、今后五年经济社会发展重点

（一）坚持科学发展，加快转变经济发展方式

把贯彻落实国家宏观调控作为发展的重大机遇，实现工作指导的战略性转变，切实抓好结构调整、加大优质投入、自主创新、节能环保四个关键环节，加快转变经济发展方式，促进又好又快发展。

狠抓结构调整优化。坚定不移地抓好产业结构调整，是实现又好又快发展的关键。坚持“抓二带一促三”和“抓三带一促二”相结合，以先进制造业为龙头，以现代服务业为支撑，以现代农业为基础，以大项目建设为重点，加快构建更具竞争力的现代产业体系。突出工业的主导地位，继续抓好大企业集团，引导骨干企业在做大的基础上，进一步做强、做稳，做到可持续发展；着力抓好中小企业发展，大力发展民营经济，形成大企业顶天立地、中小企业铺天盖地的格局。同时，积极调整工业内部结构，发挥资源和产业基础优势，拉长产业链条，培育一批核心竞争力强的企业和产品，变原料输出为产品输出，变低端产品为高端产品，变聊城制造为聊城创造。加快调整服务业结构，充分发挥区位、人文、资源优势，依托江北水城的品牌，大力发展以旅游度假业和商贸物流业为主的服务业。积极调整农业结构，着力优化种植业结构、农林牧渔业结构和农村经济结构，进一步拓宽农民增收渠道。

切实加大优质高效投入。当前，投资仍是实现聊城又好又快发展的重要支撑，要继续加大优质高效投入，以每年100个重点项目为抓手，坚定不移地上好项目、上大项目，始终保持适度的投资力度。同时，进一步优化投资结构，着重投向节能环保、科技含量高、附加值高、市场前景广阔的项目，投向先进制造业、高效农业、现代服务业和社会事业项目，投向有利于增加地方财政收入、扩大社会就业和增加城乡居民收入的项目。今年要大力抓好祥光阴极铜、信发聚氯乙烯、奥博特铜深加工、中冶银河高档文化用纸、马颊河生态文化休闲度假等项目的建设，争取聊济一级公路、500千伏输电线路工程年内开工，进一步搞好邯济铁路复线、德范高速、临高高速、聊泰高速、莘县盐化工等项目的前期准备，并储备一批大项目。

大力推进科技自主创新。把创新作为转变经济发展方式的基础，加快建设创新型城市。大力发展高新技术产业，加快用高新技术和先进适用技术改造传统产业，大力引进、培育电子信息、新材料、生物工程等高新技术产业。坚持走开放式发展科技之路，继续深化与高校院所的合作。抓好“双20”工程，鼓励企业与高校院所共建技术研究中心和国家重点实验室。今年争取新增国家级技术中心1家、省级3家，省级工程技术研究中心2-3家。集中力量抓好电动客车等“863”相关项目及其他高新技术产业化项目，力争每年新增省级以上高新技术企业10家以上。加快建设市高新技术产业园、高新技术创业服务中心、聊城软件园、科技专家工作站。大力实施“1142”信息化发展战略，建设“数字聊城”。逐年增加市县两级财政研发投入，全市研发投入占生产总值的比重逐步达到1.3%。引导企业加大科技开发投入，重点企业的技术研发经费逐步达到销售收入的3%以上。深入实施人才强市战略，高度重视知识产权保护，调动科技人员发明创造的积极性。

突出抓好节能环保。把节能减排作为科学发展的突破口和硬任务，到2010年，全市单位生产总值能耗、取水量比2005年降低23%和40%，化学需氧量和二氧化硫排放量减少到4.7万吨和8.47万吨。突出抓好50家高耗能企业的节能降耗、130家重点排污企业的污染物减排工作。大力实施“2320”工程，培育2个循环经济型县（市、区）、3个循环经济型园区、20家循环经济型企业。抓好电力工业“上大压小”工作，“十一五”期间关停小火电机组23.3万千瓦。推广节水型农业，推行城市分类供水、中水回用，建立完善用水、用电累进加价制度。加强土地管理，发展节能省地型住宅，加大推广新型墙材和禁用实心粘土砖工作力度。督促各类园区和企业搞好存量土地的挖潜利用，鼓励建设多层厂房，提高单位土地利用效率和集约化程度，开发区和各工业园区每亩投资强度要分别达到150万和100万元以上。牢固树立生态文明理念，强化“生态是借贷而不是继承”的意识，积极推进生态市、生态县、生态示范区建设。加强环保监管和执法，建立完善的污染源自动检测体系，严格执行环境影响评价和“三同时”制度，坚决落实“一票否决”和“四个不批”。实施农村小康环保行动计划，加大农业面源污染治理力度。今年，以“四河一湖”碧水行动为重点，搞好重点流域水污染防治，保质保量、按时完成县（市、区）5座重点污水处理厂和配套管网建设，确保正常运营。强化大气污染治理，全面完成29家电厂脱硫工程建设，大力开展植树造林，林木覆盖率力争达到29%以上。

（二）坚持三农稳市，扎实推进社会主义新农村建设

始终把三农工作作为重中之重来抓，以中央20字方针为指导，以增加农民收入为中心，以调整优化产业结构、发展产业化经营、深化综合改革为重点，加快实现农业大市向农业强市转变。

实施六项兴农工程，发展现代农业。一是结构优化工程。在严格保护耕地、稳定粮食供求的基础上，以更开放的思路、更有力的措施，放手发展蔬菜、食用菌、花卉等高效经济作物、林牧渔业和农村二三产业，力争到2012年，粮经比调整到5∶5，林牧渔业占农业总产值的比重达到50%，农村二三产业比重达到60%。二是农业产业化工程。增加农业投入，改革投入方式，优化发展环境，创新发展模式，大力发展各种形式的农业产业化龙头企业，把加工增值的效益留在农村，力争到2012年国家级龙头企业达到4家、省级30家、市级200家，农产品加工增值率提高到1.8。下大力推动农业标准化、国际化，完善农产品安全标准体系和监督体系，确保农产品质量安全，五年新增国家级农业标准化示范基地30个，新增“三品”认证100个，农产品出口年均增长25%。三是粮食转化工程。切实加强优质粮食基地建设，积极发展订单农业，推行成方连片种植，努力实现优质优价，下大气力引进和培育大型粮食加工企业，到2012年小麦优质优价率达到50%，商品粮加工转化率达到80%。四是市场建设工程。要科学规划、合理布局、突出特色，加快建设一批农产品专业批发市场特别是大型综合批发市场，大力发展农村运销组织和运销大户，构建现代农村市场流通体系。今年要开工建设市级综合批发市场。五是科技兴农工程。积极探索多元化农村科技服务体系，创新科技推广体制，提高科技推广效益，推进科技特派员工程，加强多种形式的农民培训，提高农民致富能力，抓好高校毕业生到农村基层服务计划和一村一名大学生工程，积极推进农业机械化，使农村发展尽快转入依靠科技进步和提高劳动者素质上来。六是劳动力转移工程。劳务输出是聊城农民增收最重要的渠道，要发扬成绩，加大力度，力争实现新的突破，到2012年突破140万人，劳务收入占农民纯收入的比重超过50%。

加大城乡统筹力度，积极为农民群众办实事。调整财政支出结构，加大支农投入力度，建立财政支农资金稳定增长机制，不断改善农民群众的生产生活条件。在这里，我代表市政府郑重承诺，今年继续为农民办好十件实事：一是进一步改善农村交通条件，新建改造农村公路800公里，改造农村危桥146座，新建农村客运站25个；二是进一步改善农村生产条件，加强农田水利建设，加快位山、郭口、陶城铺、彭楼等灌区的续建配套和节水改造，新打更新机井3000眼，新增10万人用上自来水；三是进一步改善电力通讯条件，新增农村有线电视用户3万家，新增农村固定电话5万部、宽带7万户，改造农村中低压线路800公里；四是加快发展农村清洁能源，新增沼气用户、太阳能用户各5万家、秸秆综合利用400万吨；五是全面建立新型农村合作医疗制度，参合率力争达到95%，财政补贴标准每人提高20元；六是全面建立和完善农村最低生活保障制度和农村五保供养制度，完成60处中心敬老院建设任务，集中供养率达到70%；七是建立农村中小学校舍维护长效保障机制，重点抓好50个农村中心中小学建设项目；八是实施农村电影放映工程，每个村庄每月放映一场电影；九是加快推进“万村千乡”市场工程，标准化的“农家店”覆盖全市80%以上的乡镇和60%以上的村庄；十是大力开展农村环境综合整治，改善村容村貌，净化美化农民生活环境。

创新体制机制，全面深化农村综合改革。深入推进农村义务教育、乡镇机构和县级财政管理体制改革，提高农村公共服务水平。在稳定完善农村土地承包关系的前提下，引导和规范农村土地承包经营权流转，探索适度规模经营。加快农村小型基础设施产权制度改革，建立完善农村公路和自来水等基础设施养护管理体制。加快推进农村金融体制改革，积极引导各类金融机构增加对农业的信贷投放，引导邮政储蓄等资金返还农村。鼓励建立各类农民专业合作社，支持“一村一品、一品一社”建设，提高农民组织化水平。

（三）坚持工业强市，加快新型工业化步伐

加快推进新型工业化是聊城实现跨越发展、摆脱欠发达状态的根本出路，是解决所有矛盾和问题的核心和关键，是大力发展各项社会事业、构建和谐聊城的基础和前提。要适应新时期、新阶段的新形势、新要求，更加坚定实施工业强市战略的决心，更加注重转变发展方式，努力走出一条具有聊城特色的新型工业化道路，加快向工业大市转变。每年规模以上工业增加值增幅要达到20%以上，主营业务收入和利税增幅在30%以上。

抓好“三个突破”。一是重点企业发展的突破。大力引导和支持大中型企业尤其是“5513”工程企业深入开展技术、管理、制度创新，不断增强核心竞争力。积极发展“三高”产品，大上项目，上好项目，努力在全国同行业进三强、争第一，做百年企业。力争五年内有1家企业主营业务收入过500亿元、2家过300亿元、10家过100亿元，有1家企业进入全国百强、10家企业进入全省百强。二是集群发展的突破。坚持百亿产业发展战略，全力拉长电力及有色金属、汽车及农业装备、林浆纸业、医药、食品、化工、纺织服装等七大产业链条，大力发展轴承、钢管、钎具、电缆等特色产业，促进产业集聚，培育一批特色鲜明、优势突出的产业集群，形成既有参天大树、又有茂密森林的发展格局。三是品牌建设的突破。实施品牌带动战略，提升企业综合素质和竞争能力，鼓励优势企业参与国家乃至国际标准的制定。五年内全市95%以上的主要工业产品要采用国际标准，重点培育中国名牌产品18个、中国驰名商标10枚、国家免检产品40个，创省政府质量奖5个，1000家企业（单位）通过质量体系认证。

强化园区建设。园区是加快工业化的主要载体，搞好园区建设，一是明确目标，自我加压。按照不低于全省园区平均发展速度确定目标，增强发展的压力感，并搞好考核。二是坚持园区建设与城市发展互动，以超前的思维、开放的理念、攻坚破难的智慧解决发展中的问题，努力营造比较优势和洼地效应。三是创新发展理念，实现优势互补、错位发展。要逐步从各园区结构雷同、低水平竞争中走出来，搞好园区间产业发展的空间布局和资源整合，努力形成特色突出、各具优势、互相促进、协调发展的格局。要把提升、发展开发区放到工业发展更加重要的位置，搞好规划，创新机制，制定政策，举全市之力，努力把开发区建成以汽车制造、有色金属精深加工和电子信息、生物技术等符合国家产业政策的全市优势产业聚集区，建成全市经济发展的增长极。

破解资金瓶颈。积极搭建银企合作平台，鼓励辖内外商业银行加大信贷投放，今年，要在银团贷款方面取得新突破，解决大企业、大项目的资金需求。加大招商引资力度，特别是引导有条件的企业在寻找战略合作伙伴方面取得突破。帮助和引导企业走开资本运作的路子，通过股份改造、产权出让、债券融资，逐步扩大直接融资规模，力争今后五年境内外上市企业新增10家，其中今年新增3家。

重视企业家队伍建设。企业家是我市经济发展最稀缺、最宝贵的资源，要在政治上爱护、工作上支持、生活上关心，为企业家干事创业、贡献社会创造良好的环境和条件。

（四）坚持三产兴市，推动服务业全面快速健康发展

加快发展服务业，是提升经济素质和运行质量的重要举措，是缓解就业压力的有效途径，是实现经济社会可持续发展的必然要求。要把服务业摆在经济社会发展更加突出的位置，搞好规划，制定政策，扩大总量，优化结构，逐步提升在国民经济中的比重。

突出抓好旅游业。围绕建设全国旅游热点城市，着力促进旅游产业升级和结构优化，尽快实现观光游向休闲度假游的转变，打造国内外知名的旅游目的地。重点搞好古城区和运河两岸历史著名景观保护性开发等重大旅游项目建设，争创5A级和更多4A级旅游景区。整合全市旅游资源，挖掘景区景点文化内涵，打造精品旅游线路。加大旅游度假基础设施建设，尤其是星级酒店建设，规范提高软硬件服务层次和水平，增强对国内外游客的吸引力。争取开通“北京—聊城”汽运旅游专线及铁路客运始发列车。

大力发展商贸物流业。围绕打造山东西部物流商贸基地，着力抓好市中心商业区、铁塔商圈、运河商贸带、城市综合服务中心及开发区物流配送中心的培育和发展，促进香江商贸、烟店轴承、开发区钢管等市场上层次、上水平，力争5家专业批发市场年成交额过100亿元。大力发展第三方物流，确保五年内有6家流通企业销售收入过10亿元。全力建设聊城物流中心，吸引北方铁路等大型企业入驻。今年要加快“四路一枢纽”、保税物流中心和粮食物流基地建设，构建现代物流大通道，增强城市发展活力。

积极发展文化产业。深化文化体制改革，完善文化产业发展政策，把文化与城建、经贸、体育、旅游结合起来，培育具有自我发展能力的大型文化企业集团，形成知名文化产业品牌。坚持走市场化运作的路子，推动文化产业发展壮大。适应国家推行带薪年休假制度和节日假期调整的新形势，探索办好节庆活动的新思路、新机制、新办法，把节庆活动办出特色。围绕扩大江北水城的影响力，适时举办世界水城论坛、“国际武训助学奖”评奖活动和农机装备、轴承、阿胶等博览会，打造水城会展品牌。大力发展体育产业，积极举办各种国际国内赛事，重点围绕沿湖沿河举办龙舟、皮划艇、垂钓、自行车、轮滑等比赛，打造运动之城、健康之城、阳光之城。

大力发展金融保险业。优化金融生态环境，支持银行业改革，加快争取辖外金融保险证券和投资机构入驻聊城，大力发展高新技术产业风险投资，规范发展农村信用合作事业，推动我市上市公司参股组建新的地方金融机构。另外，要积极发展房地产、餐饮休闲、信息中介等各项服务业。

（五）坚持城建靓市，进一步提升城市规划建设管理水平

城镇化是加快新型工业化进程的强大支撑，是促进服务业快速发展的主要载体，是实现以城带乡、从根本上解决三农问题的必然途径。要坚持打造江北水城不动摇，以“两城一河”为重点，大力提升城市品牌，不断增强城市的影响力、辐射力、带动力。

高起点定位。坚持“人为城市之本、文为城市之魂、水为城市之源、绿为城市之美”的理念，着眼于现代化、生态型、园林式、卓越秀美江北水城的城市定位，进一步提升城市品位、打造城市品牌。

高水平规划。没有一流的规划，就不会有一流的城市。必须下大气力、舍得投入，聘请国内外一流专家，做一百年不落后的规划。要切实增强规划的前瞻性和科学性，搞好与国家主体功能区、产业发展、土地利用、重大基础设施等规划的衔接，优先安排关系经济社会发展的重大项目，优先安排与广大市民生活密切相关的项目。编制城镇体系和组团式城市群发展规划，完成“十二五”城市建设和景观控制等专项规划。今年要及早出台城区高层建筑区布局规划和古城保护性开发、开发区发展提升、徒骇河风情带、物流中心、马颊河生态文化休闲度假基地等重点区域的详细规划编制，完成15%的乡镇驻地详细规划和全部中心村、20%的基层村建设规划。

高标准建设。严格市场准入管理，完善招标投标、工程监理、竣工验收制度，强化质量监管，落实节能环保等各项建设标准，着力打造城市精品工程。一是搞好古城区保护性开发。运用市场手段，完善基础设施，挖掘文化底蕴，有选择地恢复历史古迹，努力打造“水中古城”新品牌。二是搞好徒骇河风情带开发。坚持景区建设与两岸开发同步规划、同步实施，逐步建成风格多样、特色鲜明的世界运河主题公园，丰富江北水城内涵。三是搞好城中村改造。制定优惠政策，创新体制机制，调动各方面的积极性，加快城中村改造步伐，力争五年基本完成城中村的整体并迁、改造，使城市面貌特别是居民区面貌发生大的改观。今年重点启动和搞好湖河水系完善、交通设施配套、城区绿化提升、城中村改造、古城区保护开发、徒骇河风情带建设、火车站改造、体育公园建设、城市综合服务中心建设等重点工程，打造江北水城新靓点。

高效能管理。树立以人为本的理念，创新城市管理体制机制，把建立完善城市管理综合执法体系、严格执法与便民利民服务有机结合起来，提高市民满意度。树立改革创新效能的理念，进一步完善市政、环卫、绿化“三位一体”城市管理新模式，引入市场竞争机制和现代管理手段，变花钱养人为花钱干事，提高城市管理效益和水平。继续创建国家环保模范城和国家园林城，切实巩固创建国家卫生城成果，下大力搞好城市绿化亮化美化和各项公共服务，为广大市民创造良好的工作生活环境。

同时，要着眼长远，构筑城乡统筹发展格局，搞好县城和中心镇建设，引导农村居民逐步向小城镇转移，加快城镇化进程。

（六）坚持改革开放，努力增强经济社会发展活力

改革是经济社会发展的强大动力，要紧紧抓住影响经济社会发展的突出矛盾和问题，创新体制机制，在一些关键领域和重要环节取得实质性突破。一是加快国有资产管理体制和国企改革。建立国有资产经营预算制度，加强国有资产监管，建立重大决策失误和财产损失责任追究制度。按照“依法改、彻底改、改彻底”的原则，加快推进国有企业改革改制和国有资产优化重组，建立规范的现代企业制度。要探索建立破产企业退出机制和退出通道，依法做好企业破产处置和职工安置工作。努力推动东阿阿胶、鲁西化工、中通客车实现整体上市。二是加快推进财税体制改革。围绕推进基本公共服务均等化，完善公共财政体系。深化部门预算改革，强化预算监督和管理，规范国有资本收益上缴和使用。从今年起，市县两级全面实行国库集中收付。完善财政奖励补助政策和县乡财政管理体制，力争三年内县乡财政得到根本好转。强化税收执法，推进综合治税和财税增收信息化工程，提高税收征管质量和效益。三是推进行政事业单位改革。着力推进行政管理体制改革，加大机构整合力度，精简和规范各类议事协调机构及其办事机构。建立完善公务员管理信息系统，全面推行编制实名制和公示制管理。加快事业单位分类改革，推动行业协会的改革与发展。采取果断措施，坚决遏制机构升格、扩编进人的势头。

民营经济日益成为国民经济的主体、骨干企业的摇篮和打造产业集群的主力。要以“全市平均每年每个乡镇增加民营经济税收300万元，固定资产投资过亿元”为目标，制定政策，优化环境，突出特色，增强活力，实现民营经济新发展、新突破。大力实施“双百工程”、“中小企业成长计划”，扶持民营企业做大做强。认真抓好融资担保、信用评价、技术服务、人才培训、法律服务、行业协会等民营企业服务体系建设。创新金融服务，大力推广华夏银行中小企业动产质押等物流金融新模式。抓好银河培训、蓝色证书培训、“百千万”培训工程，积极推行职业经理人制度。到2012年，实现民营税收100亿元，年均增长20%以上，今年要力争达到47亿元，增长23%。

努力扩大对内对外全方位开放。一是加大招商引资力度。坚持聊城以外都是外，突出招商引资重点，抓好日韩、欧美和港台、长三角、珠三角等国家和地区的招商，着力引进投资过十亿元的大项目、世界500强和高新技术项目。创新招商引资方式，充分发挥企业主体作用，紧紧围绕优化经济结构、延长产业链条、寻找战略合作伙伴，开展委托招商、网上招商、商会招商、节会招商。进一步改善投资环境，建立完善项目促进落地机制，提高招商成功率。力争今后五年全市招商引资到位资金过千亿元，今年引进投资过亿元的项目20个以上，投资千万元以上的项目到位资金过100亿元。努力扩大利用外资规模，重点抓好香港华润、韩国希杰的增资扩股和美国德壹、韩国首尔休闲集团等投资项目的建设，积极争取国际金融组织和外国政府贷款。二是加快转变对外贸易增长方式。优化出口产品结构，实施出口品牌战略。巩固日韩、东南亚市场，扩大欧美市场，大力开拓欧盟、中东市场。在抓好“5513”工程企业产品出口的同时，努力抓好中小企业和外资企业的产品出口。扩大汽车轮毂、环保节能发动机、子午线轮胎等机电产品、高新技术产品出口。提高广交会、华交会参展水平，鼓励更多的企业参加国际知名展览会。引导企业运用人民币升值后带来的价格优势，扩大紧缺资源、先进技术和关键设备进口。三是大力发展区域合作。抓住我省建设省会城市群经济圈、实施“一体两翼”发展战略，我市加入环渤海经济联合市长联席会和北京承办2008年奥运会的战略机遇，主动出击，广泛合作，加快发展。四是积极实施“走出去”战略。鼓励有条件的企业加大对国外矿产、木材、橡胶等资源和能源的开发投资，为企业做大做强做持久奠定坚实基础。继续抓好对外承包工程和外派劳务工作。认真做好外事工作，加强与友好城市的往来与合作，不断谱写对外合作的新篇章。

（七）坚持和谐建设，促进社会全面发展

提高人民生活水平、实现社会和谐是经济发展的目的和归宿。要按照民主法治、公平正义、诚信友爱、充满活力、安定有序、人与自然和谐相处的总要求，统筹发展各项社会事业，着力解决事关群众切身利益的问题，不断改善人民群众的生产生活条件，努力促进人的全面发展。

优先发展教育事业。全面贯彻落实《义务教育法》，继续把发展农村义务教育作为重点，认真落实“两免一补”政策，促进九年义务教育均衡发展。推广杜郎口中学教改经验，大力推进素质教育。继续调整中小学布局，均衡教育资源，普及高中阶段教育，到2012年，全市小学生、初中生入学率均达到100%，残疾儿童入学率不低于95%，高中阶段入学率达到90%。抓好中小学和特殊教育学校仪器配套设施建设，到2010年达到国家必配标准。重视发展学前教育，大力发展职业教育，加快推进资源整合，理顺管理体制，提高职业院校办学水平和质量，支持聊城职业技术学院快速发展。努力为聊城大学创造宽松的办学环境。鼓励规范社会办学，积极发展民办教育，多渠道增加教育投入。

高度重视卫生、文化等各项社会事业发展。健全覆盖全市城乡的疾病预防控制体系，提高突发公共卫生事件应急处置能力。加快建立覆盖城乡的基本卫生保健制度，推进城市医疗服务体系改革，抓好社区卫生服务体系建设，五年新建改建城市社区卫生服务中心19处、服务站118所，其中，今年新建服务中心10处、服务站50所。大力繁荣文化事业，加快建立覆盖城乡、普遍均等、方便有效、持续发展的公共文化服务体系，规划建设一批功能齐全、规模适当、配套完整的现代化文化基础设施。实施文化“精品工程”，整合演出资源，打造精品演出剧目。支持拍摄《海源阁》、《傅斯年》等电视、电影剧目,创作特色文化精品。加大国家级民间艺术之乡申报力度，重视和加强各类文物和非物质文化遗产的挖掘保护利用。大力开展全民健身活动，增强竞技体育实力，办好第二届市运会。今年重点搞好第十一届全运会聊城分赛场建设和筹备工作。扎实做好人口与计划生育工作，稳定低生育水平，优化出生婴儿性别结构，完善流动人口管理服务体系，大力提高出生人口素质。注重国防建设，深入开展双拥共建，确保实现“全国双拥模范城”三连冠。高度重视人口老龄化问题，积极发展老龄事业。关心支持妇女儿童、残疾人事业，进一步做好广播电视、新闻出版、民族、宗教、对台、侨务、人防、档案、史志、气象、测绘、防震减灾等各项工作。

努力扩大就业。把充分就业作为经济社会发展的重要目标，确保每年新增城镇就业和再就业不低于6万人。要全面落实各项就业扶持政策，建立促进就业增长的长效机制，重点解决零就业家庭、“4050”下岗人员、失地农民的就业再就业问题。大力发展创业培训、职业培训，提高失业人员和进城务工农民的创业、就业能力。切实维护劳动者的合法权益，建立和谐的劳动关系。各位代表！收入是民生之源。今后五年，我们要着力提高低收入者收入，逐步提高工人最低工资标准，不断提高干部职工的工资水平，让更多群众拥有财产性收入，确保广大干部群众共享经济发展成果！

切实加强社会保障体系建设。以扩大社会保障覆盖面和提高保障能力为重点，全面推进社会保障制度改革。做实企业基本养老保险个人账户，完善城镇职工基本医疗保险和失业、工伤、生育保险制度。今年启动城镇居民医疗保险，搞好新型农村养老保险试点。全面推行城乡低保、医疗救助制度，确保应保尽保。更加重视关心社会弱势群体，建立健全城乡低收入困难群众的社会救助体系。做好防灾减灾和优抚安置工作。完善五保财政供养制度，推进敬老院、社区老年服务机构、儿童福利机构建设，鼓励社会慈善、社会捐赠、群众互助等社会扶助活动。

加快推进安康居住工程。积极调整住房供应结构，重点推进中低价位、中小套型普通商品住房建设，切实增加住房有效供应，控制房价过快上涨。优先发展经济适用房和廉租住房，五年新增建筑面积97.5万平方米，其中今年新建20万平方米。每年免费为农村贫困残疾人建设新居1万平方米。加快建立健全城市低收入家庭住房保障体系，力争到2010年，基本解决人均住房建筑面积10平方米以下低收入家庭住房困难。

深入推进平安聊城建设。强化社会治安综合治理，完善治安防控体系，依法打击犯罪和境内外敌对势力的各种破坏活动，深入开展与“法轮功”等邪教组织的斗争，维护国家安全和社会稳定。健全矛盾纠纷排查调处机制，正确处理人民内部矛盾，认真做好人民群众来信来访。高度重视并处理好征地、房屋拆迁、企业改制、环境保护中涉及群众切身利益的问题。严格落实安全生产责任制，强化基层基础工作，建立“科技、制度、监管”三位一体的安全生产保障体系，严防各类重大恶性事故的发生。抓好食品药品安全整治工作，建立长效机制，确保产品质量和食品安全。

加强精神文明和民主法制建设。大力弘扬社会主义核心价值体系，发扬“自强不息、务实创新、包容开放、诚实守信”的聊城人文精神，深入开展群众性精神文明创建活动，倡导和谐理念，培育和谐精神，不断提高人民群众的文化素质和社会文明程度。积极推动“诚信聊城”建设。大力弘扬法治精神，营造政治文明、法制健全的良好环境，保障人民享有广泛的民主权利。各级政府要自觉接受人大及其常委会的监督，认真听取人民政协在民主监督中的意见和建议，广泛听取民主党派、群众团体等各方面的意见,真正做到科学决策、民主决策、依法决策。认真办好人大代表议案、建议和政协委员提案。积极推进社区建设,搞好村民自治,促进城乡基层民主政治建设。充分发挥民间组织的积极作用，增强社会自治功能。完善基层民主管理制度，进一步推行政务公开、厂务公开、村务公开。深入开展普法宣传，加强法律援助工作，提高全民法律意识和全社会的法制管理水平。

四、全面加强政府自身建设

（一）坚持依法行政，着力提高政府的执行力。进一步解放思想，更新观念，增强落实科学发展观和正确政绩观的自觉性、坚定性，巩固解放思想大讨论活动成果，着力增强政府的执行力和公信力。严格依照法定权限和程序行使权力、履行职责，依法管理经济社会事务。健全重大事项社会公示制度、社会听证制度、专家咨询制度，完善决策评估和责任追究制度，推进决策的科学化、民主化、制度化。强化行政执法责任制，加强行政复议和执法监督。健全完善考核评价体系，认真考核，严格奖惩，坚决兑现。坚持真抓实干，在抓落实上狠下功夫，大力开展政府绩效评估，坚决破除本位主义，确保令行禁止、政令畅通。全面落实行政问责制，对各种行政不作为和乱作为坚决查处，决不姑息！

（二）加强学习研究，切实改进工作作风。面对新的形势，各级政府及其工作人员必须坚持与时俱进，深入学习中国特色社会主义理论，保持政治上的坚定性。认真学习现代经济、科技、社会管理、法律等知识，不断丰富头脑，提高驾驭全局的能力。进一步加强调查研究，大兴学习之风、研究之风、议政之风，把学习和研究工作结合起来，坚持理论联系实际，努力提升工作水平。牢固树立执政为民的理念，脚踏实地为群众办实事、解难事、做好事。大力发扬求真务实的优良作风，坚决反对形式主义、官僚主义，坚决反对弄虚作假、虚报浮夸。

（三）推进职能转变，努力营造春天般的发展环境。发展的竞争，关键是环境的竞争；环境的竞争，很大程度上取决于政府的效能。要深入推进行政审批制度改革，进一步减少行政审批，加强行政服务大厅建设，简化办事程序，明确审批时限，提高办事效率。继续推进政务公开，加强政府网站建设，大力发展电子政务，提高政府工作的透明度。加强社会管理和公共服务，增强基本公共服务能力，着力解决人民群众反映强烈的问题。健全市、县、乡三级突发公共事件应急管理体系，提高政府保障公共安全的能力。严格控制和规范对企业的检查评比，对乱收费、乱罚款、乱摊派行为，一经发现，从严惩处！对严重破坏聊城发展环境、造成恶劣影响的地方和单位主要负责人，坚决依法依纪进行查处!

（四）狠抓廉政建设，深入开展反腐败斗争。各级政府及其工作人员要认真遵守国家法律，坚决贯彻执行中央和省、市委关于廉洁从政的各项规定，切实加强反腐倡廉建设。按照“标本兼治、综合治理、惩防并举、注重预防”的方针，进一步完善惩治和预防腐败体系，拓展从源头上防治腐败的工作领域。深入开展反腐倡廉教育，筑牢拒腐防变思想道德防线。始终牢记“两个务必”，坚决反对铺张浪费。深入治理商业贿赂，加大腐败案件查处力度。认真落实廉政建设责任制、领导干部任期经济责任审计和述职述廉制度，严格履行廉政承诺，以身作则，抓好班子，带好队伍，树立良好的政府形象。

各位代表！聊城正处在跨越发展的关键时期。全面建设小康社会是历史赋予我们光荣而艰巨的任务，是全市人民的热切期盼。让我们更加紧密地团结在以胡锦涛同志为总书记的党中央周围，在省委、省政府和市委的坚强领导下，团结一心，开拓奋进，实现科学发展、和谐发展、跨越发展，为加快建设强市名城、创造聊城更加美好的未来而努力奋斗！
